# Whether niche changes promote the evolution of species: a case study of *Paeonia* in Asia and North America

**DOI:** 10.3389/fpls.2024.1413707

**Published:** 2024-11-15

**Authors:** Yihui Wang, Yuan Chen, Zeljana Prijic, Tatjana Markovic, Yingmin Lyu, Caihuan Tian, Xiuxin Zhang

**Affiliations:** ^1^ Beijing Key Laboratory of Ornamental Germplasm Innovation and Molecular Breeding, China National Engineering Research Center for Floriculture, College of Landscape Architecture, Beijing Forestry University, Beijing, China; ^2^ State Key Laboratory of Vegetable Biobreeding, Key Laboratory of Biology and Genetic Improvement of Flower Crops (North China), Institute of Vegetables and Flowers, Chinese Academy of Agricultural Sciences, Ministry of Agriculture and Rural Affairs, Beijing, China; ^3^ Chongqing Academy of Agricultural Sciences, Chongqing, China; ^4^ Institute for Medicinal Plants Research “Dr Josif Pančić”, Belgrade, Serbia

**Keywords:** phylogenetic relationship, Paeoniaceae, *Paeonia*, niche change, niche

## Abstract

Ecological changes have been observed to promote rates of lineage diversification, yet the precise roles of ecological factors, species evolution, and environmental variability in driving species diversity remain research hot spots. The association between ecological change and lineage diversification, particularly with regard to the size of the time scale, remains poorly understood. To explore whether ecological change facilitates species evolution, we focused on the unique family Paeoniaceae, which encompasses both herbaceous and woody taxa, to investigate the evolutionary rates. As a unique family characterized by a single genus of angiosperms and comprising various climatic types, the ecological niche changes of *Paeoniaceae* are closely associated with the evolution, making it an ideal model for conducting association analysis. In this study, we integrated the molecular fragments and ecological factors to explore the relationship between species evolution and niche changes in Paeoniaceae. The phylogenetic tree revealed that Paeoniaceae forms a sister relationship with *Penthoraceae*, *Haloragidaceae*, *Iteaceae*, *Crassulaceae*, and *Saxifragaceae*, constituting an independent clade based on the positive selection of molecular fragments including two protein-coding genes and eight non-coding regions. The divergence time was estimated to be between 102 and 116 Mya (Million years ago). The phylogenetic tree within *Paeonia* revealed a clear division into three groups: sections of *Paeonia*, *Moutan*, and *Onaepia* with high support values for each branch based on the ten positive selection of molecular fragments. The rapid rate of evolution observed in *Paeonia*, about 0-5 Mya. In addition, ecological niche modeling showed that the potential distributions for *Paeonia* expanded from middle Asia to eastern Asia, and from central North America to the Northern part of North America during the Last Glacial Maximum (LGM) to Mid Holocene (MID) period. This suggests that *Paeonia* continuously adapted to changing ecological environments over time. Compared to the rate of climatic niche divergence and lineage diversification, the ecological niche of *Paeonia* underwent significant changes during the period of 3-11 Mya, occurring 5 Mya earlier than the period of evolutionary rate change. These findings offer comprehensive insights into the relationship between niche change and the evolution of species, providing valuable perspectives for further ecological cultivation efforts.

## 1 Introduction

Evolution is a long-term process influencing species diversity, regulated at a macroscopic level by climate change and at a microscopic level by nucleotide variation (Pyron and Burbrink, 2013). For microevolution, the inferences regarding adaptive evolution within DNA sequences serve as research hot spots for biologists (Chen et al., 2021). The divergence time of species, as determined by plastid super-barcodes such as *ndh*H, *rpl*23, and *rbc*L, is extensively utilized (Krawczyk et al., 2018; Wu et al., 2021a). In recent years, with advancements in sequencing technology, the utilization of the entire chloroplast (cp) genome has emerged as a versatile tool for phylogenetics (Dong et al., 2018; Zhai et al., 2019; Raman and Park, 2016). Indeed, some studies have shown that the recognition of pervasive adaptive evolution, based on high levels of genetic diversity, relies on positive selection of molecular fragments (Shapiro et al., 2007; Yang et al., 2005). Signals of positive selection could accurately assess the divergence time between species (Tajima, 1989; Fay and Wu, 2000; Sabeti et al., 2002; Chen et al., 2021). It has previously been reported that *Cem*A, *rps*7, and *rpl*23 genes exhibit relatively higher nucleotide substitution rates than other genes within Poaceae and Typhaceae (Guisinger et al., 2010). Similar patterns were observed within Saxifragales. In addition, *ycf*1 and *ycf*2 emerged as the predominant genes with high nucleotide substitution rates in many plants, including Caryophyllaceae (Sloan et al., 2012), Geraniaceae (Guisinger et al., 2011) and *Prunus* (Xue et al., 2019). Given their extensive coding lengths, *ycf*1 and *ycf*2 are prone to frequent substitution rate variations (Neubig et al., 2009; Huang et al., 2010; Dong et al., 2015).

For macroevolution, aspects of climate change influence the distribution of angiosperms globally and the clade of species for evolutionary processes (Haffer, 1997; Barnagaud et al., 2012; Ali and Aitchison, 2014). Studies indicate that climate and niche change had a profound impact on the diversity of plant species in Eastern Eurasia since the Last Glacial Maximum (Song et al., 2024). Additionally, the global diversity of angiosperms has undergone significant changes due to Quaternary climate and niche fluctuations (Xu et al., 2023). Clearly, climate changes are closely related to species distribution. However, the impact of natural selection on species unfolds over extended periods, leaving the question of whether ecological niches change during species evolution still open for debate (Laland et al., 1996; Laland and Feldman, 1999; Wiens and Graham, 2005; Losos, 2008). In recent years, studies on niche changes and species evolution have been increased. Broadly speaking, the periods of rapid diversification in angiosperms are similar to the periods of rapid changes in average annual temperature and precipitation (Liu et al., 2020). The average diversification rate of angiosperm niches is comparable to the rapid divergence time of the Crassulaceae based on 301 protein-coding loci (Folk et al., 2019). Therefore, there is a universal need for studies investigating the relationship between changes in ecological niches and the evolutionary history of species.

Paeoniaceae Raf. is a family of angiosperms, comprising a single genus and 34 species, including both herbaceous and woody plants found across Asia, Europe, and North America (Hong, 2010, 2021). This taxon exhibits diverse habitats in temperate conditions, including Monsoon Climate, Temperate Continental Climate, Temperate Marine Climate, and Plateau Climate. Its distribution spans three continents, North America, Asia, and Europe (Hong, 2010, 2021), indicating a rich diversity of ecological environments. The Paeoniaceae has been classified within Saxifragales based on molecular data (Dong et al., 2018; Folk et al., 2019; Li et al., 2019, 2021), diverging from its previous placement within Ranunculidae based on morphological characteristics (Guan, 1979). The bootstrap and posterior probability values of the Paeoniaceae clade are relatively low due to the limited diversity of genetic fragments (Li et al., 2019, 2021). In comparison with the findings in the literature, there exists a significant disparity in the estimated divergence times of Paeoniaceae based on chloroplast genomes or molecular fragments (Li et al., 2019; Zhou et al., 2021). Therefore, the selection of appropriate molecular fragments is vital for determining both the systematic placement of Paeoniaceae within Saxifragales and the accurate estimation of its divergence time.

Among the 34 species of Paeoniaceae, 15 species are distributed in Asia and North America (Hong, 2010, 2021). Despite efforts to estimate the divergence times of *Paeonia* in Asia based on fossils from other families within Saxifragales (Zhou et al., 2021; Chen et al., 2023), the phylogenetic position of Paeoniaceae within Saxifragales remains uncertain. To explore whether ecological changes drive species evolution, it is urgent to study the relationship between ecological niche change and divergence times of *Paeonia*. However, only the ecological niche of *Paeonia rockii* has been studied thus far (Dong et al., 2022).

In this study, we adopted an approach that utilizes a positive selection of molecular fragments from whole chloroplast genome sequences to comprehensively infer phylogenetic relationships and divergence times within Paeoniaceae and other related families. Besides, we explored the change in ecological niches and the evolutionary history of *Paeonia* in Asia to elucidate whether niche changes facilitate species evolution. Our study offers novel insights into niche dynamics within a family encompassing species evolution.

## 2 Materials and methods

### 2.1 Sequence data

We obtained sequence data for 294 species within Saxifragales and outgroups from the National Center for Biotechnology Information (NCBI: https://www.ncbi.nlm.nih.gov/). These data are detailed in **Supplementary Table S1**. The dataset included species from the following families: Paeoniaceae (29), Hamamelidaceae (38), Altingiaceae (10), Cercidiphyllaceae (2), Daphniphyllaceae (5), Saxifragaceae (108), Grossulariaceae (5), Iteaceae (1), Crassulaceae (79), Haloragidaceae (7), Penthoraceae (1), and outgroups (9). The Genbank numbers sourced from NCBI are listed in **Supplementary Table S1**.

### 2.2 Positive-selection signals screening and genome comparison

To estimate rates of nucleotide substitution, we calculated the ratio of the number of nonsynonymous substitutions per nonsynonymous site (Ka) to the number of synonymous substitutions per synonymous site (Ks) using the YN model in KaKs_Calculator (Zhang, 2022) for the protein-coding genes (PCGs). We employed the genetic code with 11 bacterial, archaeal, and plant plastid codes (Yang and Nielsen, 2000) for the species obtained from NCBI (**Supplementary Table S2**). The ratio of non-coding nucleotide substitution rate (Kn) to neutral substitution rate (Ks) was determined for the non-coding sequences by comparing aligned adjacent coding sequences. Notably, the genetic code used remains the 11-bacterial, archaeal, and plant plastid codes (Yang and Nielsen, 2000). Subsequently, we generated plots of Ka/Ks and Kn/Ks using the R-packages ‘tidyverse’ and ‘readxl’ (Wickham and Lionel, 2019; Hadley and Bryan, 2019). To investigate plastid genome divergence, we computed the nucleotide diversity (Pi) for the 74 common protein-coding genes and 72 common non-coding regions using DnaSP v5 (Librado and Rozas, 2009). Next, we identified hotspots characterized by high numbers of nucleotide substitutions and sequence distance (Pi) and then generated Pi plots using R-packages ‘tidyverse’ and ‘readxl’ (Wickham and Lionel, 2019; Hadley and Bryan, 2019).

### 2.3 Phylogenetic analysis within Saxifragales

In order to infer phylogenetic relationships within Paeoniaceae and other related families, we obtained 294 high-quality chloroplast genome data and comprehensive annotation files from NCBI (**Supplementary Table S1**). Given that the important lineages within Saxifragales consist of the clade of Saxifragaceae, Penthoraceae, Haloragidaceae, Crassulaceae, Iteaceae, and Grossulariaceae, we selected 201 representative species. As the focus of this study, we selected all 29 published data within Paeoniaceae, and selected 64 species to represent the other clade within Saxifragales and outgroups. Based on the 294 sequence data, we utilized MAFFT version 7.49 to compare the sequence fragments matrix (including *ycf*1, *ycf*2, *ndh*D-*psa*C, *trn*C-*pet*N, *trn*K-*rps*16, *ycf*4-*cem*A, *trn*H-*psb*A, *pet*G-*trn*W, *pet*N-*psb*M, and *pet*A-*psb*J) using the ‘–auto’ strategy and normal alignment mode (Katoh and Standley, 2013). Ambiguously aligned fragments were subsequently removed using Gblocks version 0.91b with the following parameter settings: minimum number of sequences for a conserved/flank position (10/10), maximum number of contiguous non-conserved positions (8), minimum length of a block (10), and allowed half of the gap positions (Talavera and Castresana, 2007). For the species tree, a maximum likelihood (ML) phylogeny for the genera was inferred using RAxML v8.0.0 (Stamatakis et al., 2008). The best-fitting DNA substitution model for the ML tree was determined to be GTR (General Time Reversible) + F (Felsenstein) + I (proportion of Invariable sites) using ModelFinder (Kalyaanamoorthy et al., 2017). A standard bootstrap with 5000 replicates is used to infer the ML tree. In addition, Bayesian inference (BI) analysis was conducted using MrBayes version 3.2.6 (Ronquist and Huelsenbeck, 2003), with the best-fitting DNA substitution model being GTR + F + I. Markov chain Monte Carlo simulations (MCMC) were run for 10,000,000 generations. The BI analysis commenced with a random tree and sampled trees every 1,000 generations. The first 25% of the trees were discarded as burn-in, and the remaining trees were used to generate a majority-rule consensus tree. Subsequently, the trees were edited using ITOL to display bootstrap (BS) and posterior probability (PP) values (https://itol.embl.de/; Letunic and Bork, 2021).

### 2.4 Molecular dating within Saxifragales

In BEAST 1.10.4, a lognormal distribution with an uncorrelated relaxed clock model was run using the GTR + F + I site model, with a random starting tree and a Yule speciation process tree prior (Yule, 1927; Suchard et al., 2018). MCMC was performed for 500 million generations, with samples taken every 50,000 generations. The effective sample size (ESS) values were verified to exceed 200 for all parameters. The initial 25% of the trees were designated as burn-in and discarded. The remaining trees were then used to generate an all-compatible consensus tree using TreeAnnotator software (Drummond and Rambaut, 2007). Moreover, 95% highest posterior density intervals (HPD) for each node are shown on the tree. Besides, the phylogeny was calibrated using five fossils. The first calibration point was set to the age of Saxifragaceae (81 Mya with HPD about 72.1-89.8 Mya; Bell, 1957). The second calibration point was the age of Hamamelidaceae (77 Mya with HPD of about 70.6-83.5 Mya; Itterbeeck et al., 2007). The third calibration point was the age of Altingiaceae (91 Mya with HPD of about 89.3-93.5 Mya; Zhou et al., 2001). The fourth calibration point was the age of Cercidiphyllaceae (68.3 Mya with HPD of about 66-70.6 Mya; Brown, 1962). The last calibration point was the age of Vitales (123 Mya with an HPD of about 101.8-144.1 Mya; Li et al., 2019). The tree was viewed and edited using ITOL and FigTree version 1.4.0 (https://itol.embl.de/; http://tree.bio.ed.ac.uk/software/Figtree/; Letunic and Bork, 2021).

### 2.5 Phylogenetic analysis and molecular dating within Paeoniaceae

The phylogenetic analysis within the Paeoniaceae family was conducted using 15 wild species as listed in **Supplementary Table S1**. The analysis was based on a matrix comprising 10 sequence fragments and was performed using both the ML and BI methods utilizing RAxML v8.0.0 and MrBayes version 3.2.6 (Ronquist and Huelsenbeck, 2003; Stamatakis et al., 2008). Among the 15 species, *Chrysosplenium album* (NC067019), *Heuchera abramsii* (MN496062), and *Saxifraga sikkimensis* (NC070509) were designated as outgroups. The parameters used for MAFFT version 7.49, Gblocks version 0.91b, RAxML v8.0.0, and MrBayes version 3.2.6 were consistent with those employed for the phylogenetic analysis within Saxifragales (Ronquist and Huelsenbeck, 2003; Talavera and Castresana, 2007; Stamatakis et al., 2008; Katoh and Standley, 2013). The best-fitting DNA substitution model for both methods was determined to be GTR + F + I. Given the absence of reliable fossils within Paeoniaceae, we initially used the estimated time frame of 106-112 Mya, as previously described. Next, we used the 70.6-83.5 Mya divergence as the calibration point for Saxifragaceae (Bell, 1957). The parameters employed in BEAST 1.10.4 and TreeAnnotator software were consistent with those utilized in the phylogenetic analysis within Saxifragales (Drummond and Rambaut, 2007; Suchard et al., 2018). Finally, the ‘ape’ R package was used to calculate and plot the evolutionary rate of *Paeonia* (Paradis and Schliep, 2019).

### 2.6 Geographic distributions of species

We acquired distribution data of the 15 species distributed in Asia and North America listed in **Supplementary Table S1** of Paeoniaceae from various sources, including the Chinese Virtual Herbarium (http://www.cvh.org.cn/), Global Biodiversity Information Facility database (http://www.gbif.org/), Flora of China (Guan, 1979), and records from field surveys conducted by our research group. Each data about latitude and longitude information for each data point was meticulously verified, and specimens lacking such coordinates were reconstructed based on microclimate data. A total of 9,702 record points were screened and included in the analysis (**Supplementary Table S3**). Subsequently, we filtered and duplicated the data with similar latitude and longitude, ensuring a minimum distance of less than 5 kilometers between each point, and visualized the distribution of the dataset using the R-packages ‘dplyr’ and ‘geosphere’ (Karney, 2013; Morgan and Cheng, 2023).

### 2.7 Climate data screening and filtering

We obtained climate data for two historical periods comprising 19 bioclimatic and elevation layers for the last glacial maximum (LGM, about 22,000 years) and Mid Holocene (MID, about 6000 years ago) in the WorldClim global climate database (https://www.worldclim.org/). The LGM and MID data were obtained from the CCSM4 climate model. To ensure greater accuracy about the variables, we performed the correlation analysis between individual environmental factors and species with the method of spearman and remove environment variables with low relevance (Murdoch and Chow, 1996).

### 2.8 Analysis of suitable distribution areas

The collected data, comprising distribution points and the precise variables without bio8, bio9, bio18 and bio19, were imported into MaxEnt 3.4.1 software for modeling analysis with the best model using the R package “kuenm” (Phillips et al., 2017; Muscarella et al., 2014). The 75% of the distribution points were treated as the training data, while the remaining 25% were designated as random test data. We optimized the 29 feature combinations (FC) and regularization multipliers (RM) from the “kuenm” package (ranging from 0 to 4.0 with an interval of 0.1), resulting in the generation of 2,480 candidate models. The model with the smallest delta AICc was selected as the optimal solution and used to establish the final model. Next, the final model (linear features and quadratic features) was replicated 10 times using cross-validation to assess accuracy and the output data format was logistic (Moreno et al., 2011). A regularization multiplier of 0.1 was applied, and the maximum number of background points was set to 10,000. Besides, the number of iterations for each dataset in MaxEnt maximum was set to 500. The convergence threshold for the predictions was established at 0.0001, and the probability of presence at ordinary occurrence points was set to 0.5 (Elith et al., 2011). The criterion for assessing the accuracy of the model’s predictions is based on the area under the curve (AUC) value of the Receiver Operator Characteristic (ROC) (Hebbar et al., 2022). AUC >0.7 is typically considered indicative of a good model (Rebelo et al., 2010). The determination of the main bioclimatic factors influencing the suitable distribution areas of *Paeonia* in Asia was conducted using the method of Jackknifing. Finally, we classified suitable areas into the following four categories using natural breaks (Jenks): non-suitability (p < 0.08), low suitability (0.08 ≤ p < 0.26), moderate suitability (0.26 ≤p < 0.43), and high suitability (0.43 ≤ p < 1) during the LGM period (Saleh, 2020). During the MID period, suitable areas were classified into the following four categories: non-suitability (p <0.09), low suitability (0.09≤p < 0.27), moderate suitability (0.27 ≤p < 0.46), and high suitability (0.46≤p < 1).

To explore the change in distributions between LGM and MID periods, we used the SDM_Toolbox_v2.4 package to visualize the potential variations in suitable areas (Brown et al., 2017). Initially, the potential suitable areas were converted into binary vectors, with suitability probabilities of species set at 0.43 and 0.46, respectively.

### 2.9 Rate of climatic niche divergence

To estimate the rate of climatic niche divergence in *Paeonia*, we analyzed the similarity of climatic niche spaces from each clade using the R package ‘ecospat’ (Broennimann et al., 2011; Cola et al., 2017). For each clade and the overall environmental niches from the phylogenetic tree, we compared the similarity of the environmental niches using Schoener’s D niche metric ranges, where D-Values range from 0 to 1 (Warren et al., 2008). Principal component analysis (PCA) was used to determine the proportion of environmental factors contributing to the ecological niche (Broennimann et al., 2011). Finally, we compared the rate of evolution with the rate of climatic niche divergence in *Paeonia* using the R package ‘ggplot2’.

## 3 Results

### 3.1 Nucleotide substitution rates

**Figure 1:**
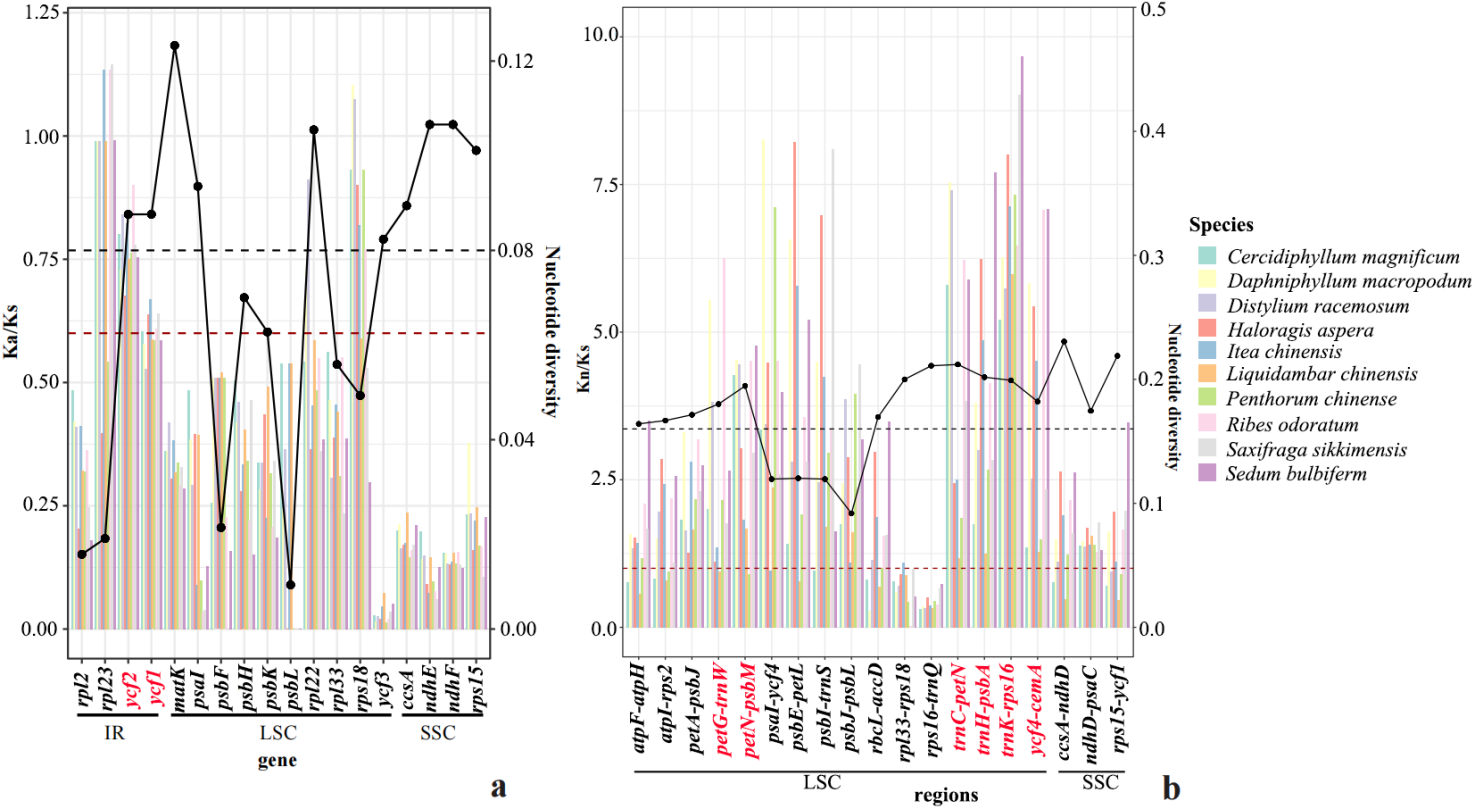
The map of Ka (Kn)/Ks and Pi analysis within 11 species. **(A)** The estimations of Ka to Ks of plastid protein-coding genes (PCG). The estimations of nonsynonymous (dN), synonymous (dS) substitution rates, and dN/dS of plastid protein-coding genes (PCG). **(B)** The estimations of Kn/Ks of plastid genes. The estimations of nonsynonymous (dN), synonymous (dS) substitution rates, and dN/dS of plastid noncoding regions.

To trace the evolutionary rates along *Paeonia*, we analyzed nucleotide substitution rates relative to neutral substitution rates on sequences, along with Pi analysis. We screened for common high nucleotide substitution rates of sequence fragments based on methods established in previous research (Zhou et al., 2021; Chen et al., 2023). First, Nonsynonymous (Ka) and Synonymous (Ks) substitution rates, along with Ka/Ks, were estimated for the 74 common protein-coding genes to detect evolutionary rate heterogeneity. Among the 74 genes analyzed, large ribosomal protein (*rpl*23), hypothetical chloroplast reading frames (*ycf*2), hypothetical chloroplast reading frames (*ycf*1), large ribosomal protein (*rpl*22), and small ribosomal protein (*rps*18) exhibited relatively higher Ka/Ks values (Ka/Ks > 0.6), suggesting they have been subjected to positive selection pressures. The remaining genes exhibited relatively lower Ka/Ks values (Ka/Ks < 0.6), suggesting their exposure to purifying selection pressure (**Figure 1A**; **Supplementary Figure S1**). We also compared Kn/Ks across non-coding regions to identify variations in evolutionary rate. Among 72 non-coding regions, we selected those with high Kn/Ks values across 11 species. Elevated Kn/Ks values (Kn/Ks > 1) had been identified within the following non-coding regions: *ndh*D-*psa*C, *trn*C-*pet*N, *trn*K-*rps*16, *ycf*4-*cem*A, *trn*H-*psb*A, *pet*G-*trn*W, *pet*N-*psb*M, *pet*A-*psb*J, *psa*I-*ycf*4, *psb*E-*pet*L, *psb*I-*trn*S, and *psb*J-*psb*L (**Figure 1B**; **Supplementary Figure S1**).

The Pi analysis identified several chloroplast regions (*atp*F-*atp*H, *atp*I-*rps*2, *ccs*A-*ndh*D, *ndh*D-*psa*C, *pet*A-*psb*J, *pet*G-*trn*W, *pet*N-*psb*M, *psb*Z-*trn*G, *rbc*L-*acc*D, *rpl*33-*rpl*18, *rps*15-*ycf*1, *rps*16-*trn*Q, *trn*C-*pet*N, *trn*D-*trn*Y, *trn*G-*trn*R, *trn*H-*psb*A, *trn*K-*rps*16, *trn*L-*trn*F, *trn*S-*trn*G, *trn*Y-*trn*E, and *ycf*4-*cem*A) and genes (*ccs*A, *mat*K. *ndh*E, *ndh*F, *psa*I, *rps*15, *ycf*1, *ycf*2, and *ycf*3) to be divergent hotspot regions (**Figure 1**; **Supplementary Figure S2**). In comparison to Pi analysis, Ka/Ks and Kn/Ks, the commonly identified potential molecular markers for species identification and phylogenetic evolution within Saxifragales include the following 10 regions: *ndh*D-*psa*C, *trn*C-*pet*N, *trn*K-*rps*16, *ycf*4-*cem*A, *trn*H-*psb*A, *pet*G-*trn*W, *pet*N-*psb*M, *pet*A-*psb*J, *ycf*1, and *ycf*2.

### 3.2 Phylogenetic relationships and divergence time estimation within Saxifragales

**Figure 2:**
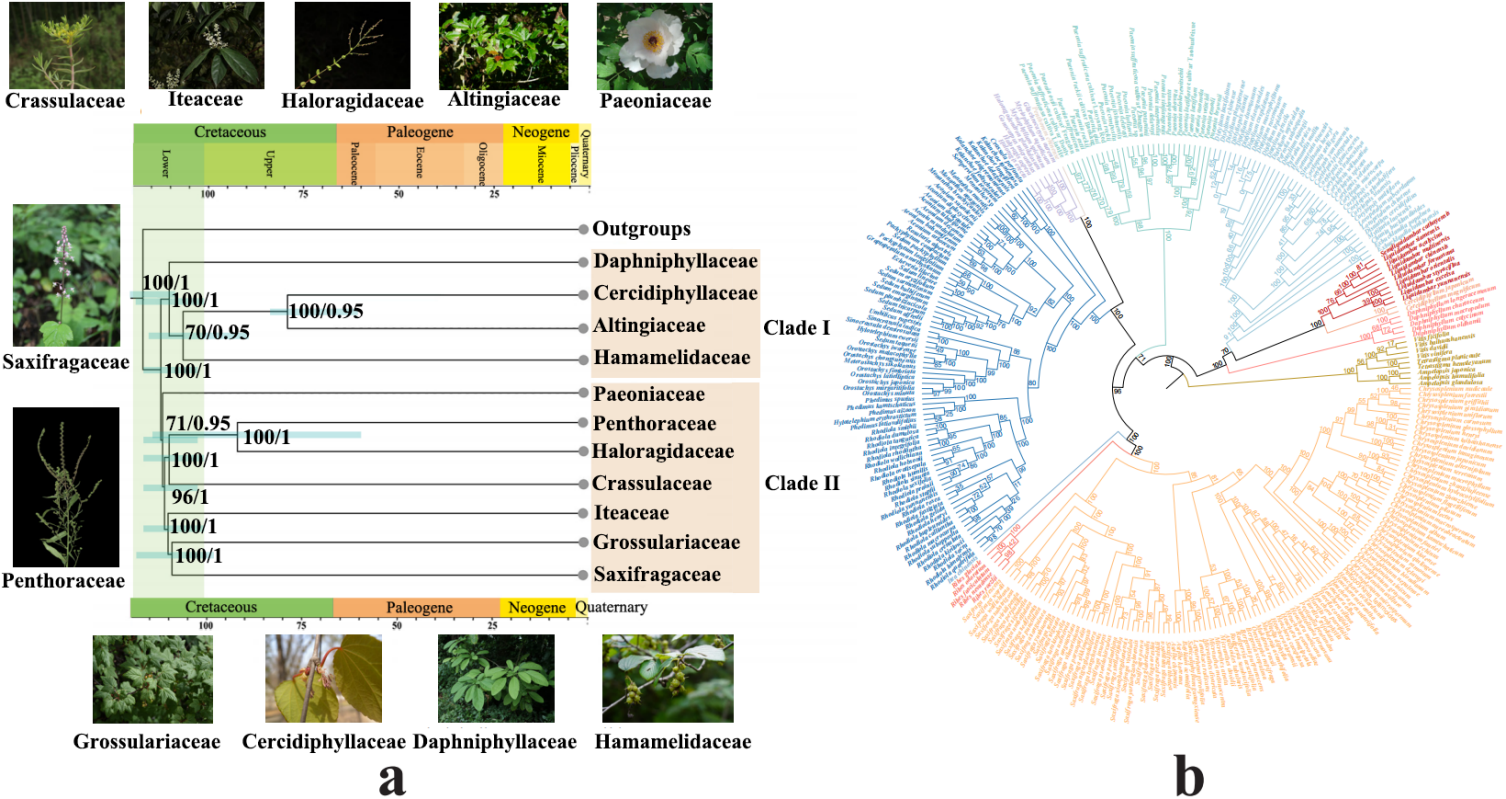
Phylogenetic and divergence time analysis within Saxifragales **(A)** Results of phylogenetic and divergence time analysis within Saxifragales. The 95% highest posterior density (HPD) credibility intervals for node ages are labelled above the line. The numbers on the clade represent the support value and posterior probability. **(B)** ML tree within Saxifragales. The numbers on the clade represent the support value.

We conducted a phylogenetic analysis to determine the systematic classification of Paeoniaceae within Saxifragales. Based on data from 10 regions extracted from chloroplast genomes, our phylogenetic reconstructions showed a similar topological tree between the ML tree and BI tree within the family clades (**Figure 2**; **Supplementary Figure S3**). The nodes within each family exhibited high bootstrap values and posterior probabilities, indicating a reliable inference of phylogenetic relationships. The tree comprehensively resolved the phylogenetic relationships within Saxifragales among the major clades. Saxifragales underwent a divergence event at approximately111 Mya (HPD:102-117 Mya), giving rise to two large sub-clades: Clade I (comprising *Hamamelidaceae*, *Altingiaceae*, *Cercidiphyllaceae*, and *Daphniphyllaceae*) and Clade II (encompassing *Paeoniaceae*, *Penthoraceae*, *Haloragidaceae*, *Iteaceae*, *Crassulaceae*, and *Saxifragaceae*) with a PP of 1 and a BS of 100. Paeoniaceae diverged from Clade II at approximately 110 Mya (HPD:102-116 Mya) forming an independent clade with a PP of 0.95 and a BS of 71. The lineages, including Penthoraceae, Haloragidaceae, and Crassulaceae, initiated diversification at 109 Mya (HPD:102-116 Mya) from the Iteaceae, Grossulariaceae, and Saxifragaceae groups, with a PP of 1 and a BS of 96. Crassulaceae, as the sister group to Haloragidaceae and Penthoraceae, underwent diversification at 108 Mya (HPD:100-117 Mya) with a PP of 1 and a BS of 100. Haloragidaceae began diversifying from Penthoraceae at 90 Mya (HPD:56-115 Mya), with a PP of 1 and a BS of 100. Iteaceae commenced diversification from Grossulariaceae and Saxifragaceae groups at 107 Mya (HPD: 100-117 Mya), with a PP of 1 and a BS of 100. Furthermore, Saxifragaceae initiated diversification from Grossulariaceae at 106 Mya (HPD: 97-117 Mya), with a PP of 1 and a BS of 100. Within Clade I, Daphniphyllaceae diverged approximately 107 Mya (HPD: 99-117 Mya) as an independent clade from Cercidiphyllaceae, Altingiaceae, and Hamamelidaceae groups, with a PP of 1 and a BS of 100. Hamamelidaceae diverged from Altingiaceae and Hamamelidaceae groups at around 104 Mya (HPD: 87-115 Mya), with a PP of 0.95 and a BS of 70. Finally, it was found that Altingiaceae diverged from Hamamelidaceae at 77 Mya (HPD:70-83 Mya), with a PP of 0.95 and a BS of 100. It was evident that the majority of species within Saxifragales underwent divergence events between 100 and 115 Mya, during the lower Cretaceous period.

### 3.3 Phylogenetic relationships and divergence time estimation within Paeoniaceae

**Figure 3:**
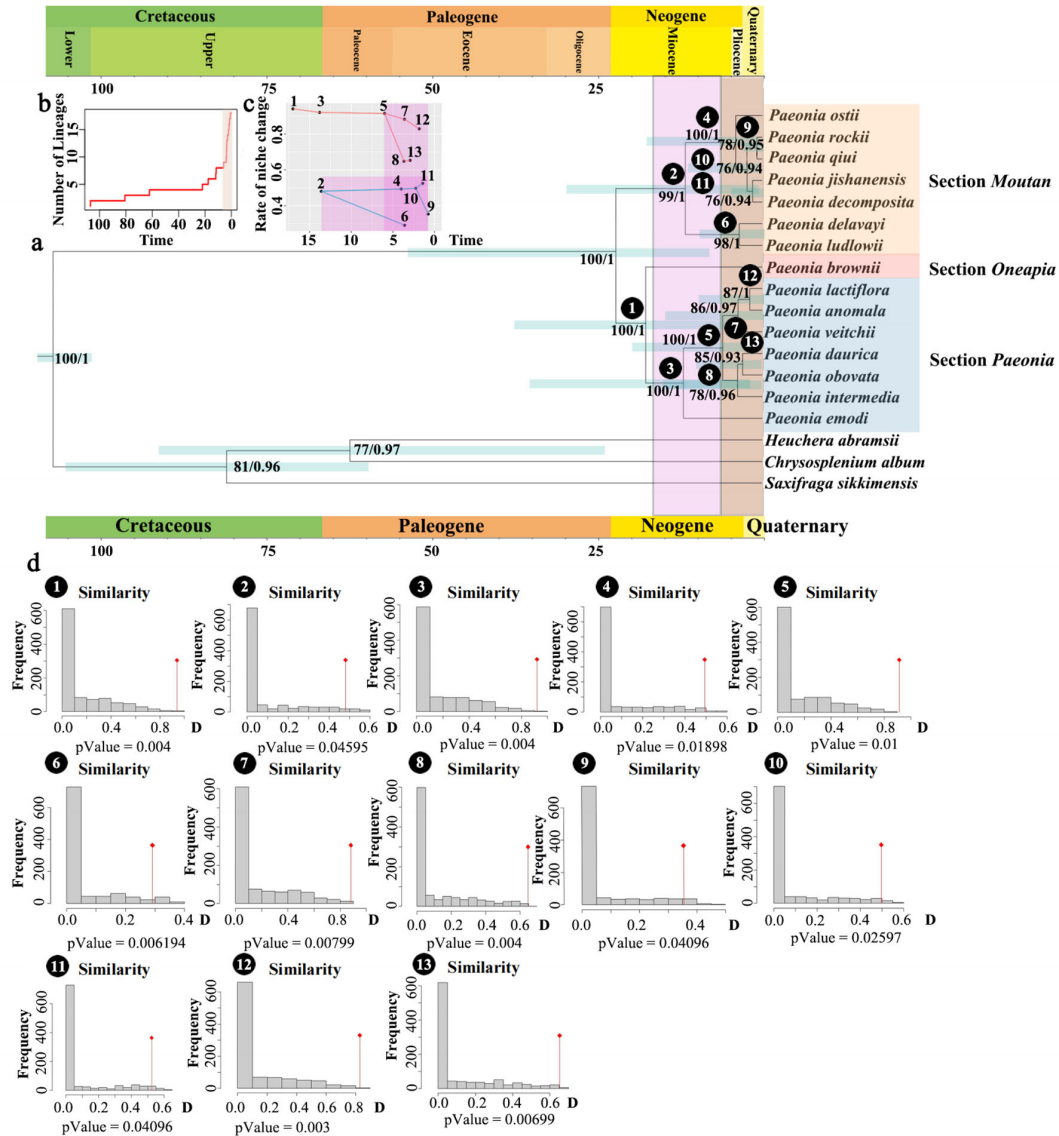
Phylogenetic and species evolution rate analysis within *Paeonia*. **(A)** Results of comparative analysis of niche changes and phylogenetic trees based on 10 regions of *Paeonia*. The 95% highest posterior density (HPD). Credibility intervals for node ages are labelled above the line. The numbers on the clade represent the support value and posterior probability. **(B)** Rate of species evolution **(C)** Rate of niche change. **(D)** Niche similarity tests.

To elucidate the evolutionary relationships within Paeoniaceae, we performed phylogenetic analyses of the genus *Paeonia* in Asia based on data from 10 regions extracted from chloroplast genomes. Notably, *Heuchera abramsii*, *Chrysasplenium album*, and *Saxifraga sikkimensis* were employed as outgroups (**Figure 3A**). The topological structures of the trees remained consistent across both ML and BI analysis methods. *Paeonia* species exhibited subdivision into three large sub-clades, corresponding to the taxonomic classification of sections of *Paeonia*, *Moutan*, and *Onaepia* (Hong, 2010, 2021). The *Moutan* section diverged from the herbaceous clades (sections of *Paeonia* and *Onaepia*) at 24 Mya (HPD:7-53 Mya), with a PP of 1 and a BS of 100. P*. brownie*, representing section *Onaepia*, formed a sister relationship with section *Paeonia* with a PP of 1 and BS of 100. This divergence occurred at approximately 18 Mya (HPD:3-36 Mya). The species in the *Moutan* section can clustered into two distinct clades: group one (*P. delavayi* and *P. ludlowii*) and group two (*P. ostii*, *P. rockii*, *P. qiui*, *P. jishanensis*, and *P. decomposita*), with a PP of 1 and a BS of 99. The divergence between the two clades occurred around 11 Mya (HPD:1-28 Mya). In section *Paeonia*, *P. emodi* was positioned at the base with a PP of 1 and BS of 100. Within this section, a subclade comprising *P. lactiflora*, *P. anomala*, and *P. veitchii* formed a sister relationship with another subclade containing *P. daurica*, *P. obovata*, and *P. intermedia* with a PP of 1 and a BS of 100. The divergence between these two subclades occurred approximately 7 Mya (HPD:1-21 Mya). Collectively, these results suggest that the rate of evolution of *Paeonia* rose rapidly from 0 to 5 Mya.

### 3.4 Potential distributions and comparisons of *Paeonia* in Asia between LGM and MID

The single-factor correlation analysis reveals that the all variables without bio8, bio9, bio18 and bio19 exhibit significant correlations with the distribution of the species (**Supplementary Figure S4**). Based on the distribution patterns of *Paeonia* in Asia and North America (**Supplementary Figure S5**) and analysis of the potential distributions during the LGM and MID periods, the mean AUC for test replicates runs 0.838 for LGM and 0.843 for MID (**Supplementary Figure S6**). Plots illustrating the contributions of respective variables were generated from a pool of 16 factors influencing distribution patterns across the two periods (**Supplementary Figure S7**). The contributions of bio11, bio12 and bio5 to the LGM period were determined to be 27.2%, 22.2% and 19.5% respectively, with a cumulative contribution of 70%. On the other hand, the contributions of bio11, bio1, bio12 and elevation, to the MID period were found to be 34.7%, 17.1%, 12.6%, and 8.5%, respectively, with a cumulative contribution of 72.9%.

**Figure 4:**
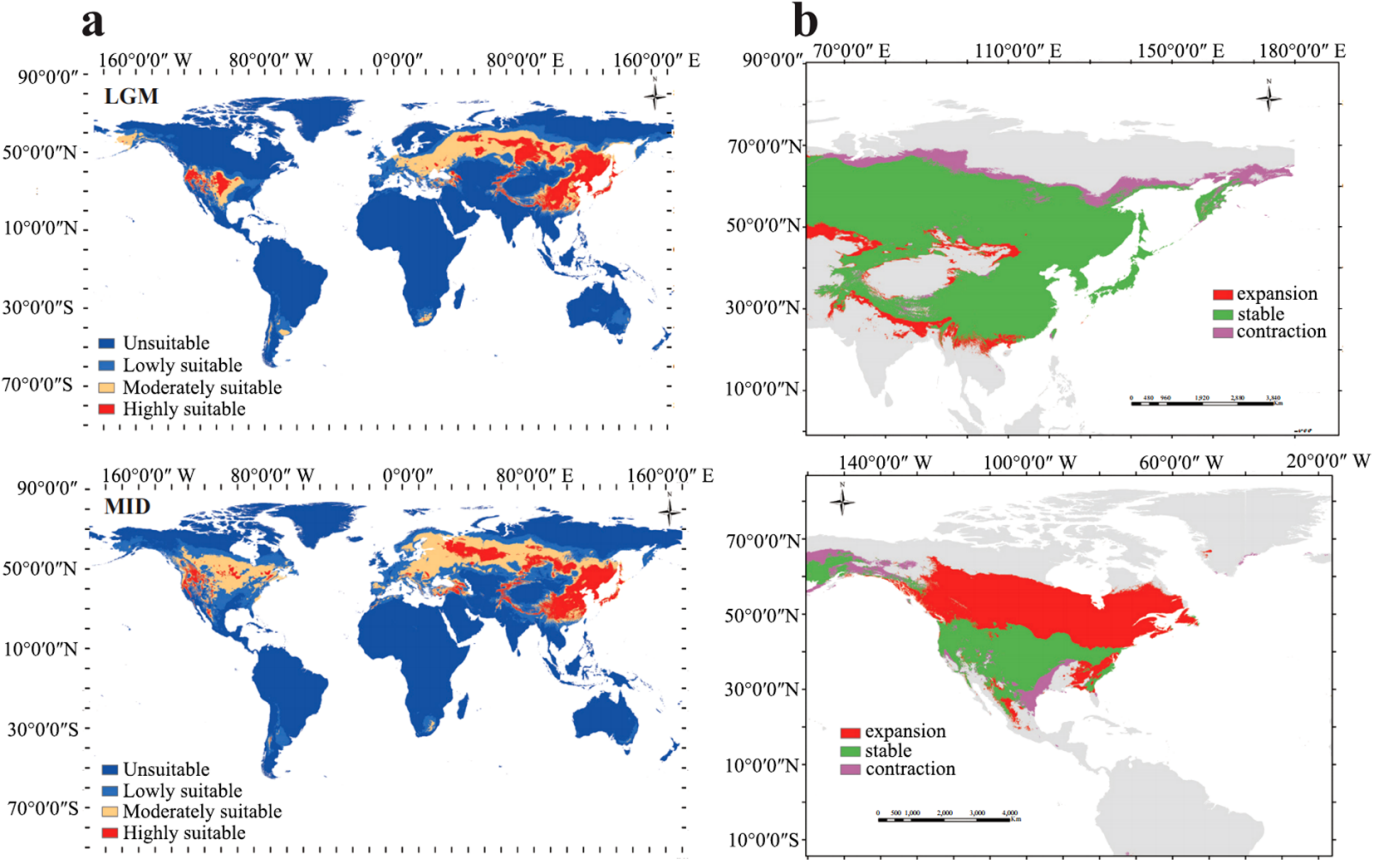
Distributions and comparisons of *Paeonia* in Asia between LGM and MID periods. **(A)** Potential distribution for *Paeonia* during LGM and MID periods using the MaxEnt model. Deep blue, blue, yellow, and red areas represent unsuitable, marginally suitable, moderately suitable, and highly suitable areas, respectively. **(B)** Spatial changes of *Paeonia* during LGM and MID periods. Gray, green, green, red, and purple areas represent unsuitable, unchanged suitable, expansion suitable, and contraction suitable areas, respectively.

Based on the natural breaks method, the potential distribution of *Paeonia* in Asia and North America during the LGM period was classified into four grades: unsuitable area, lowly suitable area, moderately suitable area, and highly suitable area. The regions identified as highly suitable for *Paeonia* distribution include central China, Japan, the Korean Peninsula, regions in middle Asia such as Russia and Kazakhstan, and North America (**Figure 4A**). The respective areas for highly, moderately, and lowly suitable distribution areas were 1079.491×10^4^ km^2^, 1658.386×10^4^ km^2^, and 1792.825×10^4^ km^2^, whereas the unsuitable area measured 10908.483×10^4^ km^2^ (**Supplementary Table S4**). For the MID period, the potential distribution of *Paeonia* in Asia and North America was also categorized into the four grades. Specifically, the regions identified as highly suitable for *Paeonia* distribution were southern China, Japan, the Korean Peninsula, middle Asia such as Russia and Kazakhstan, and western North America. The respective areas for the highly, moderately, and lowly suitable distribution zones were 1118.525×10^4^ km^2^, 2141.561×10^4^ km^2^, and 2363.145×10^4^ km^2^, while the unsuitable area measured 9811.784×10^4^ km^2^ (**Supplementary Table S4**). A comparison of the LGM and MID periods revealed that the distribution of *Paeonia* expanded from central China to southern China while contracting from northern Russia to southern Russia. Moreover, the core area of *Paeonia* extended from North America to Canada (**Figure 4B**).

### 3.5 Niche comparisons

PCA analysis was performed for niche comparisons. The findings revealed that the identified factors accounted for 99.31% of the 20 environmental factors in the distribution of *Paeonia*. PC1 accounted for 98.42%, with bio10 emerging as the main impact factor, whereas elevation and bio4 were found to be associated with PC2 (**Supplementary Figure S8**). The niche similarity, expressed as D-Value, was calculated for each main clade of the *Paeonia* tree, and the obtained results are displayed in **Supplementary Table S5** and **Figure 3B**. The plotted change in D-Value for each branch showed a significant shift in the ecological niche of *Paeonia* around 3-11 Mya, predating the period of evolutionary rate change by 5 Mya. This suggests that alterations in the ecological environment promote the evolution of species, and the time scale is 5 Mya.

## 4 Discussion

### 4.1 The positive-selection signals from plastid genomes

By estimating nucleotide substitution rates across various genes and non-coding regions, a more comprehensive understanding of species evolution can be attained (Weng et al., 2016; Schwarz et al., 2017). First, we identified two high nucleotide substitution rates of genes, ycf1 and ycf2, in the coding regions, which is consistent with the findings of Dong et al. (2015). Therefore, leveraging the information from these *ycf* genes enables more detailed profiling of the angiosperm tree of life. Moreover, in comparison with the non-coding regions, a super-barcode region (*trn*H-*psb*A) was identified, as analogous to findings in Oryza (Zhang et al., 2021) and *Dracaena* (Zhang et al., 2019). In this study, additional non-coding regions (*ndh*D-*psa*C, *trn*C-*pet*N, *trn*K-*rps*16, *ycf*4-*cem*A, *pet*G-*trn*W, *pet*N-*psb*M, and *pet*A-*psb*J) were newly identified as super-barcodes for Saxifragales. Specifically, *trn*K-*rps*16 was used to determine the systematic status of *Paeonia* in Pan-Himalayan regions (Zhou et al., 2021). Even though the regions exhibiting high nucleotide substitution rates varied when analyzed by KaKs, KnKs, and Pi methods, the commonly shared regions served as DNA super-barcodes, facilitating species delineation and the estimation of evolutionary history within Saxifragales.

### 4.2 Phylogenetic relationships and species divergence within Saxifragales

Phylogenetic analysis revealed that Paeoniaceae forms a sister relationship with the groups Penthoraceae, Haloragidaceae, Iteaceae, Crassulaceae, and Saxifragaceae. This finding is consistent with previous studies based on both coding and non-coding gene fragments (Dong et al., 2018; Zhou et al., 2021), but contradicts the results of a study focused on *atp*B, *mat*K, and *rbc*L genes (Fishbein et al., 2001). Previous studies constructing phylogenetic trees to determine the position of Paeoniaceae using molecular fragments reported lower PP and BS. Our work defined the status of Paeoniaceae and improved the value of PP and BS. Divergence time estimates for Paeoniaceae vary significantly across studies due to the utilization of different molecular fragments. For instance, reported estimates include 80-100 Mya in the Upper Cretaceous (Tank et al., 2015; Folk et al., 2019; Dong et al., 2022), 65-90 Mya in the Upper Cretaceous (Zhou et al., 2021), and 88-130 Mya in the lower Cretaceous (Li et al., 2019). The divergence of Paeoniaceae is estimated to have occurred during the lower Cretaceous at approximately 110 Mya (HPD:102-116 Mya) based on the positive-selection regions, which represents a more accurate assessment compared to previous studies. In Saxiragales, the status of other families was consistent with the majority of studies (Dong et al., 2018; Li et al., 2019; Zhou et al., 2021) and the 95% HPD interval was narrower.

### 4.3 Phylogenetic relationships and species divergence within Paeoniaceae

The inclusion of three sections of Paeoniaceae (sections *Paeonia*, *Moutan*, and *Onaepia*) within a broader evolutionary lineage garnered high BS and PP. These findings were consistent with the previous phylogenetic reconstructions (Wu et al., 2021b; Zhou et al., 2021). Furthermore, our study enhanced the BS and PP of the three main clades. We also provided evidence supporting the proximity of section *Paeonia* to section *Onaepia*, a finding corroborated by Wu et al., 2021b and Zhou et al., 2021. In addition, we subdivided section *Moutan* and section *Paeonia* into two subsections, aligning with findings from previous studies (Zhou et al., 2021; Dong et al., 2022). Regarding the divergence time, the split between woody and herbaceous *Paeonia* species was approximately 24 Mya (HPD: 7-53 Mya). This estimate falls within the range previously determined based on whole chloroplast genome sequences, which was approximately between 20.78 Mya (HPD: 12-29 Mya; Dong et al., 2022) and 28 Mya (Zhou et al., 2021). The divergence of sections *Paeonia* and *Onaepia* occurred during the Miocene period, approximately 18 Mya, consistent with previous estimates (Zhou et al., 2021). Furthermore, the rapid rate of evolution observed in *Paeonia*, about 0-5 Mya, aligns with recent research (Chen et al., 2023).

### 4.4 The change of spatial distributions of *Paeonia* in Asia and North America

Our study suggests that Paeoniaceae originated during the lower Cretaceous, potentially influenced by the warm and humid conditions prevalent in the peripheral tropical environment during the Cretaceous era (Klages et al., 2020). During the LGM period, the regions identified as highly suitable for *Paeonia* were mainly concentrated in central China, Japan, the Korean Peninsula, middle Asia including Russia and Kazakhstan, and North America. The prediction results were consistent with the actual distributions (Dong et al., 2022). Given that during the LGM period much of the Earth was characterized by extensive glaciation, resulting in a reduction of warm-tropical and subtropical habitats compared to present conditions (Clark et al., 2009), the suitable distribution areas were more concentrated. After the LGM period, as temperatures gradually increased during the post-ice age, the Earth transitioned to being predominantly covered in warm-tropical and subtropical habitats. Cool-temperate biomes were primarily confined to polar regions (Pound et al., 2012). Consequently, the suitable distribution areas expanded from Northern Asia to Southern Asia. Similar predictions have been made for other rare and endangered plants, such as *Cedrus* and *Firmiana kwangsiensis* (Xiao et al., 2022; Gao et al., 2022). Besides, the dispersal of anthers by frugivorous birds, coupled with rapid radiation evolution occurring in the early Miocene, facilitated the swift expansion of the distribution range of *Paeonia* (Moyle et al., 2016). The observed spatial distribution patterns of *Paeonia*, coinciding with climate change, are consistent with the radiation evolution of birds.

The expansion of *Paeonia* into eastern Asia can be attributed to the intensification of the East Asian summer monsoon, which occurred around the Oligocene-Miocene boundary, and was triggered by the uplift of the northern Tibetan Plateau at around 20-25 Mya (Tada et al., 2016). This resulted in the enlargement of the potential highly suitable area of Paeonia in eastern Asia. The presence of endemic *Paeonia* species in Southwest China can be verified by evidence of population isolation and formation of endemic species in the region largely due to tectonic shifts and river course dynamics throughout the Quaternary period (Qiu et al., 2011). The widespread spatial distribution of *Paeonia* can be attributed to a significant transition from a zonal distribution of arid climates to warmer and wetter climates across central and eastern Asia since the early Miocene (Guo et al., 2008). Thus, it is plausible that the intensification of the East Asian summer monsoon during the late Miocene played a crucial role in triggering species or population-level diversification within tropical regions.

### 4.5 Niche differentiation and species divergence

Utilizing 20 ecological factors, our analysis revealed compelling evidence indicating a strong positive correlation between ongoing climatic variability and species divergence within *Paeonia*. This relationship is consistent with simulation-based theoretical findings suggesting a synergistic effect between phenotypic evolution and species differentiation (Hua and Wiens, 2013), where macroevolutionary dynamics and rates are driven by ecological and microevolutionary factors, with decreasing niche width accelerating speciation diversification (Aguilée et al., 2018). Both ecological factors, specifically precipitation of the coldest quarter and temperature seasonality, exert influence on the evolutionary history of *Paeonia*, with a notable contribution to niche conservatism. Similar studies have indicated that decreasing temperature and precipitation can facilitate higher evolutionary rates (Herbert et al., 2016). Furthermore, our study suggests that the diversification rates of *Paeonia* in Asia estimated were highest during the Quaternary period, specifically around 0-5 Mya, which is consistent with findings in Theaceae (Yu et al., 2017) and evergreen oak (Jiang et al., 2016). The rates of niche change within the clade were highest at 5-11 Mya, which precedes the period of highest diversification rates. However, this result contradicts the findings of a previous study (Folk et al., 2019).

## 5 Conclusions and prospects

In this study, we used positive selection analysis of molecular fragments, including *ndh*D-*psa*C, *trn*C-*pet*N, *trn*K-*rps*16, *ycf*4-*cem*A, *trn*H-*psb*A, *pet*G-*trn*W, *pet*N-*psb*M, *pet*A-*psb*J, *ycf*1, and *ycf*2 to investigate the phylogenetic relationships within Saxifragales and Paeoniaceae. The phylogenetic results showed that Paeoniaceae formed an independent clade, and was a sister to Penthoraceae, Haloragidaceae, Iteaceae, Crassulaceae, and Saxifragaceae, with an estimated divergence time of 102-116 Mya. In addition, we inferred the ecological niche and spatial distributions of *Paeonia* in Asia and America, revealing that the potential habitat of species expanded to the southwestern edge of Asia and northern America. Moreover, the rates of niche change within the clade were observed to precede the diversification rates, occurring about 5 Mya. This study offers valuable insights into the evolution of Paeoniaceae and enhances our understanding of the relationships between evolutionary history and ecological niche change.

## Data Availability

The datasets presented in this study can be found in online repositories. The names of the repository/repositories and accession number(s) can be found in the article/**Supplementary Material**.

## Supplementary material

Supplementary Figure 1 The estimations of Ka/Ks and Kn/Ks analysis within 11 species. **(A)** The estimations of Ka/Ks of all plastid protein-coding genes (PCG). **(B)** The estimations of Kn/Ks of all plastid noncoding regions.

Supplementary Figure 2 The estimations of Pi analysis within 11 species. **(A)** The estimations of nonsynonymous (dN), synonymous (dS) substitution rates and dN/dS of all plastid protein-coding genes (PCG). **(B)** The estimations of nonsynonymous (dN), synonymous (dS) substitution rates and dN/dS of all plastid noncoding regions.

Supplementary Figure 3 BI tree within Saxifragales. The numbers on the clade represent the Posterior probability.

Supplementary Figure 4 The single-factor correlation analysis of 20 variables. The number of * symbols represents the magnitude of the P-value (* for P-value <= 0.05, ** for P-value <= 0.01, and *** for P-value <= 0.001).

Supplementary Figure 5 The map of the collection and distribution of the *Paeonia* genus in Asian and North American.

Supplementary Figure 6 The average test of AUC.

Supplementary Figure 7 The contributions of the corresponding variable were plotted from 16 factors that affected the distributions of two periods.

Supplementary Figure 8 Principal component analysis (PCA) in the ecological niche of 20 environmental factors.
