# Supplementary figures and images for: Whether niche changes promote the evolution of species: a case study of Paeonia in Asia and North America
Source: Front Plant Sci. 2024 Nov 15;15:1413707. doi: 10.3389/fpls.2024.1413707 (PMC11604445; doi:10.3389/fpls.2024.1413707)

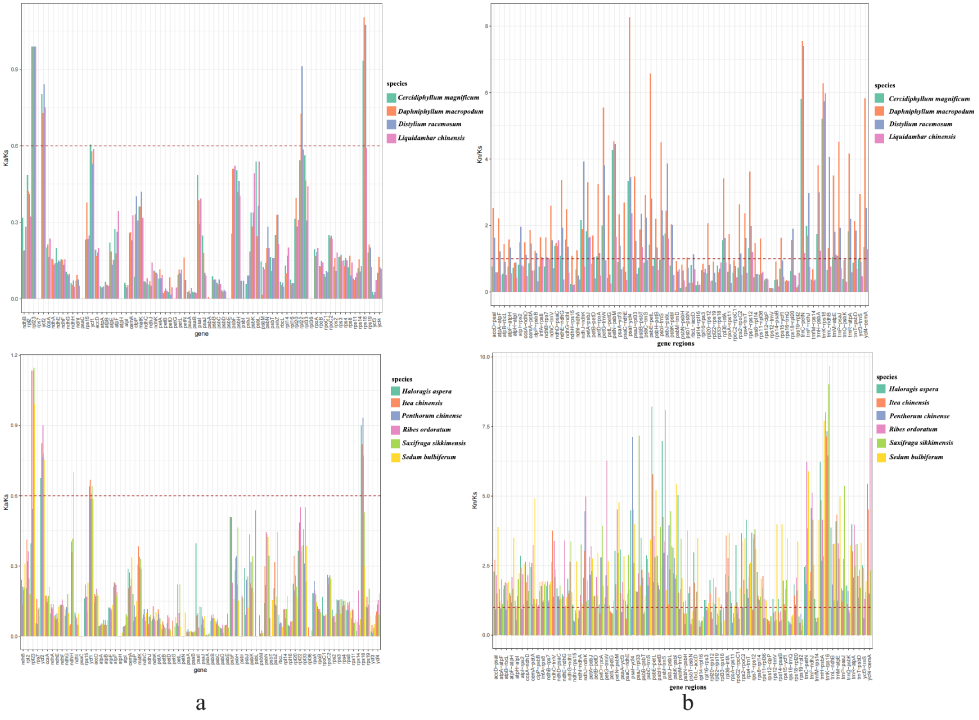

Supplement: Supplementary Figure 1 — The estimations of Ka/Ks and Kn/Ks analysis within 11 species. (A) The estimations of Ka/Ks of all plastid protein-coding genes (PCG). (B) The estimations of Kn/Ks of all plastid noncoding regions. [file Image1.tif]

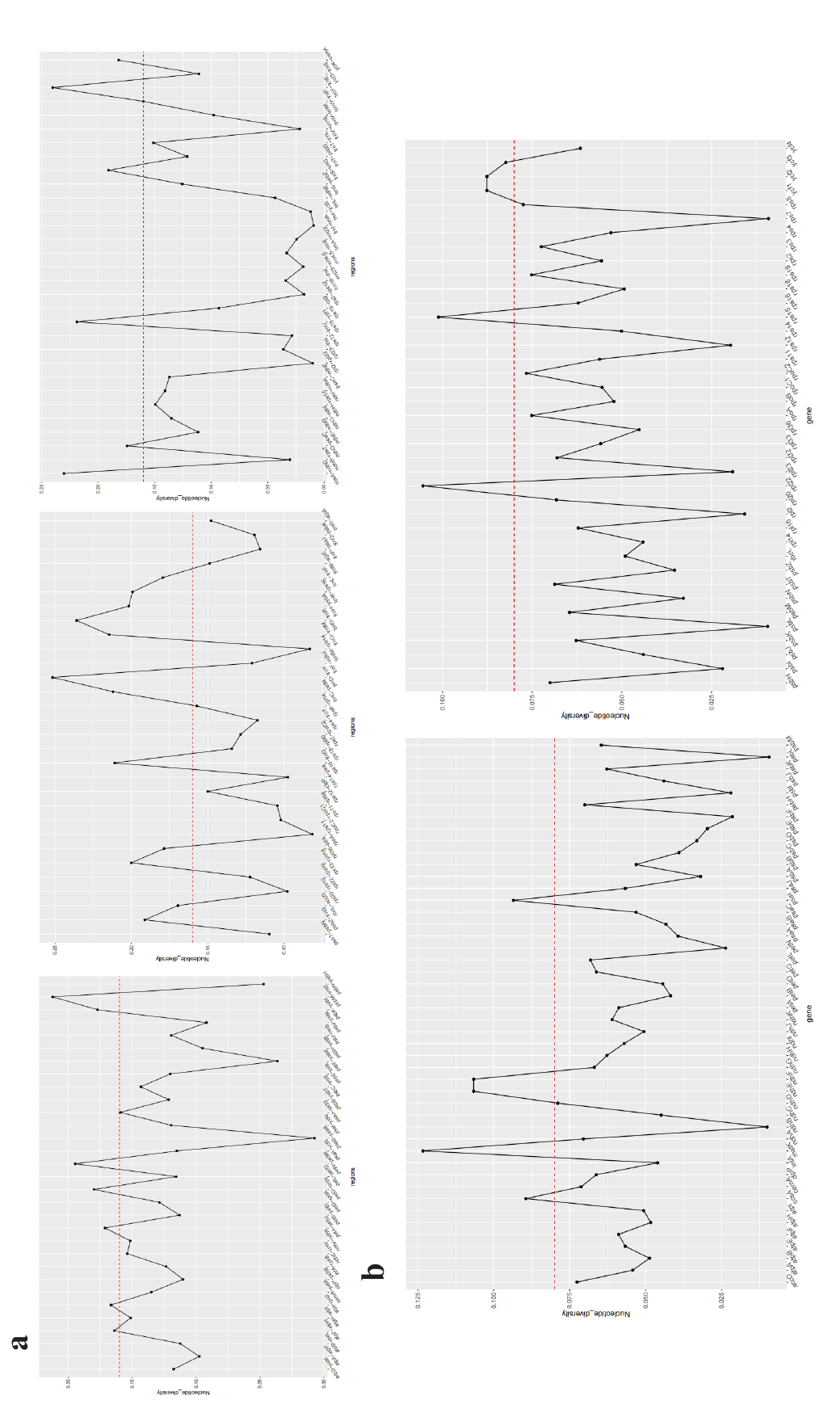

Supplement: Supplementary Figure 2 — The estimations of Pi analysis within 11 species. (A) The estimations of nonsynonymous (dN), synonymous (dS) substitution rates and dN/dS of all plastid protein-coding genes (PCG). (B) The estimations of nonsynonymous (dN), synonymous (dS) substitution rates and dN/dS of all plastid noncoding regions. [file Image2.tif]

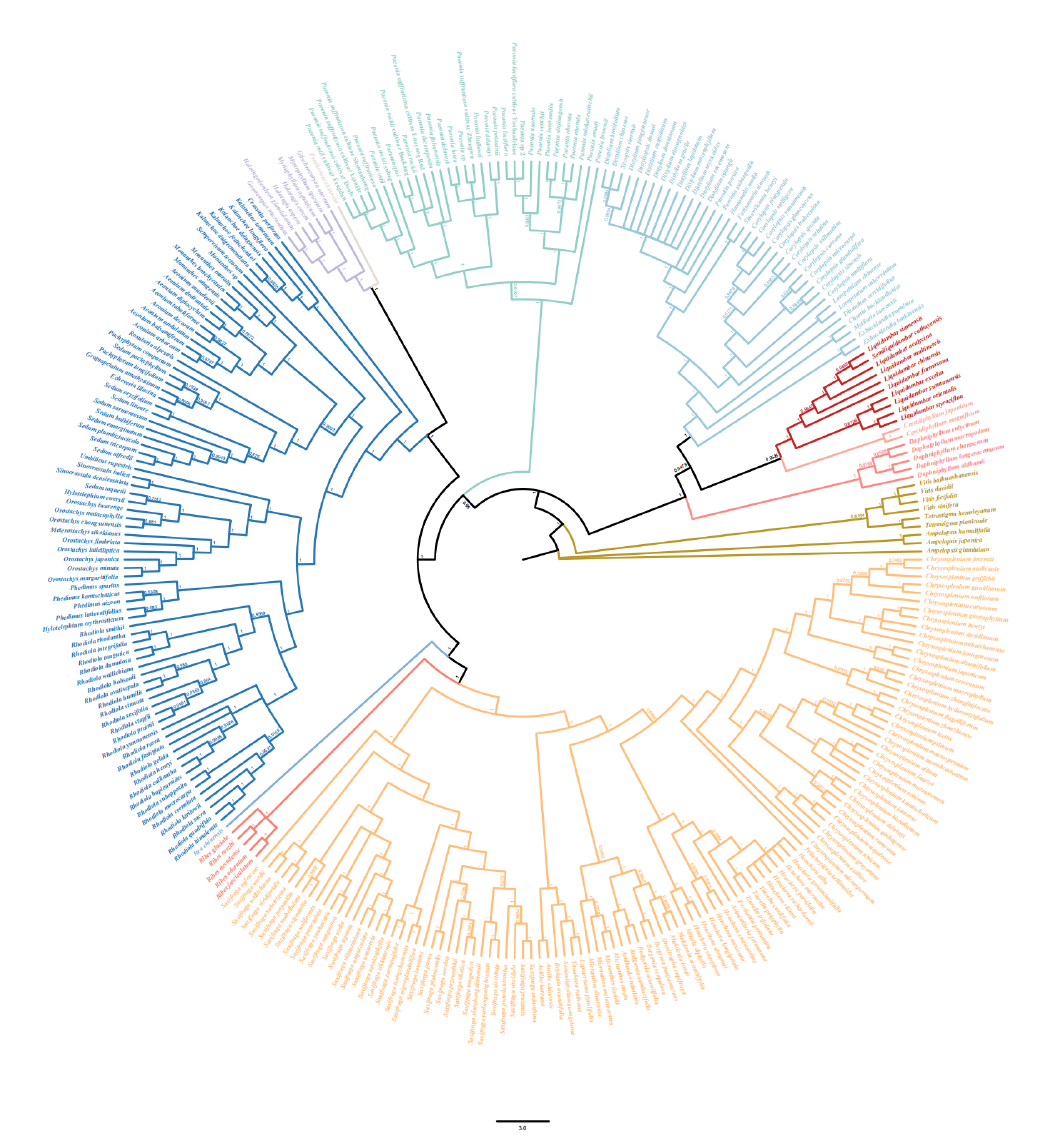

Supplement: Supplementary Figure 3 — BI tree within Saxifragales. The numbers on the clade represent the Posterior probability. [file Image3.tif]

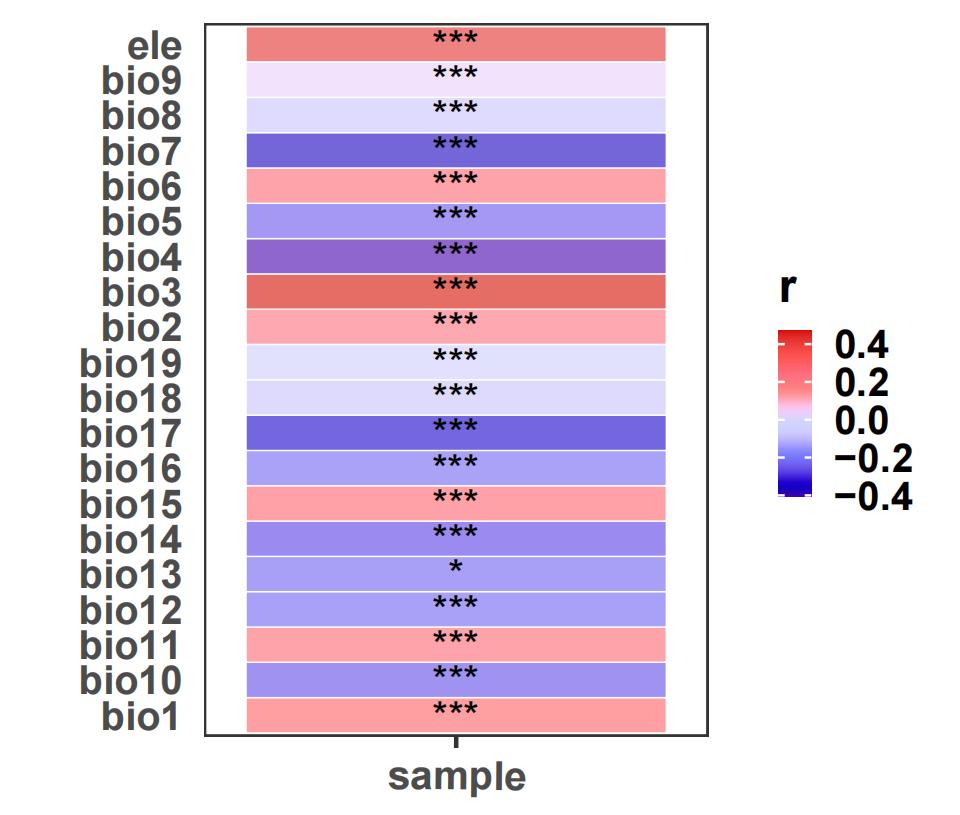

Supplement: Supplementary Figure 4 — The single-factor correlation analysis of 20 variables. The number of * symbols represents the magnitude of the P-value (* for P-value <= 0.05, ** for P-value <= 0.01, and *** for P-value <= 0.001). [file Image4.jpg]

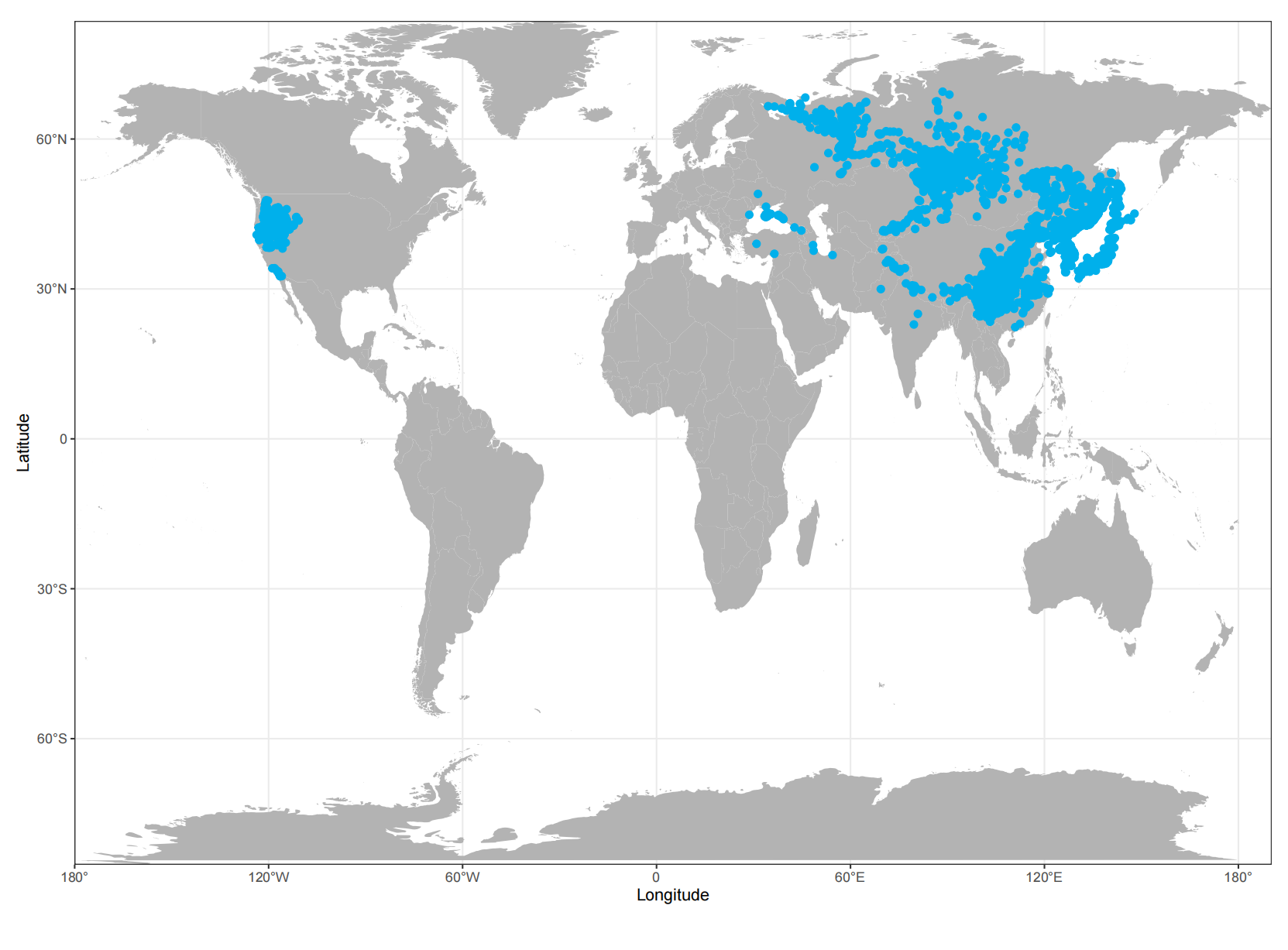

Supplement: Supplementary Figure 5 — The map of the collection and distribution of the Paeonia genus in Asian and North American. [file Image5.tif]

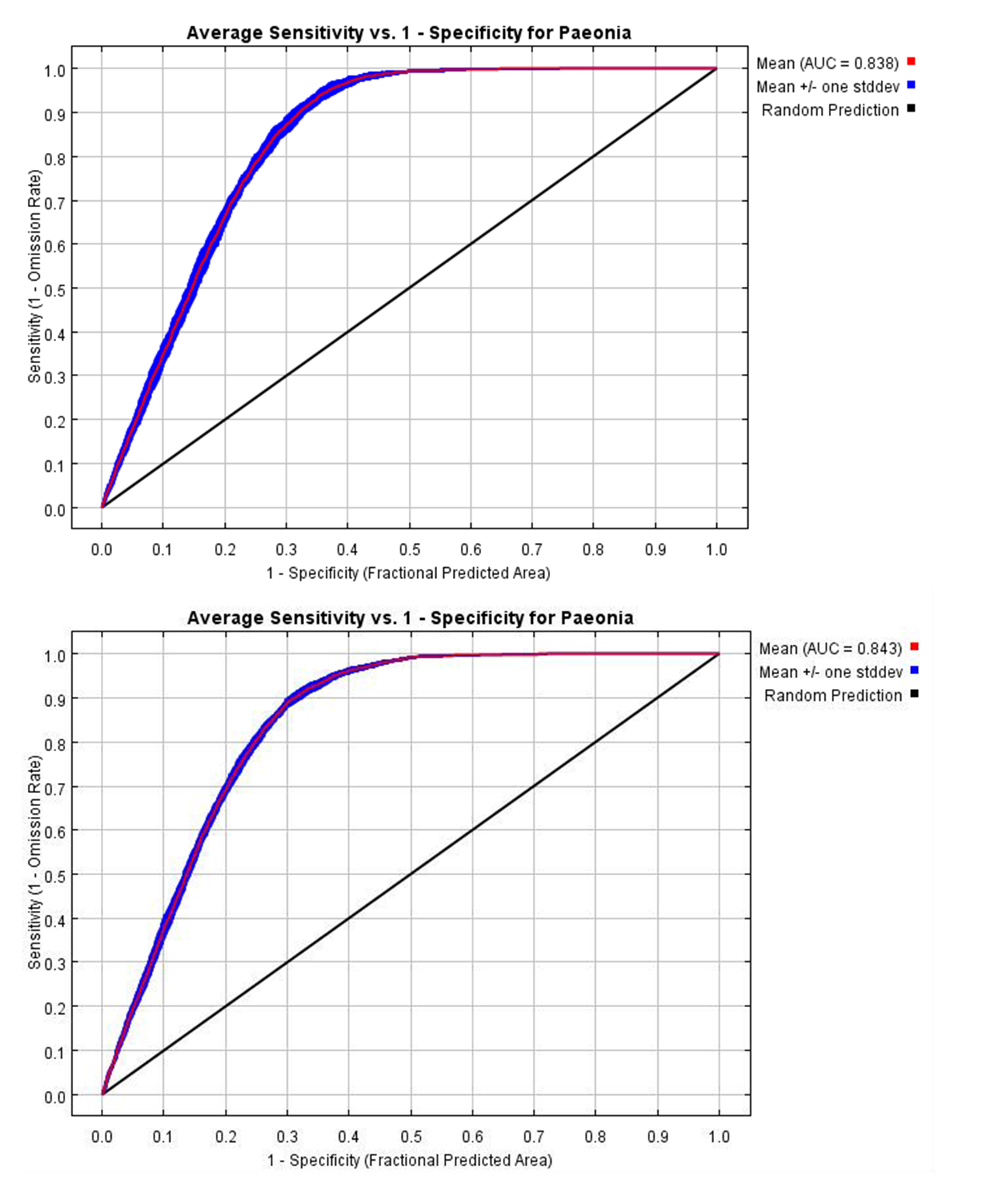

Supplement: Supplementary Figure 6 — The average test of AUC. [file Image6.tif]

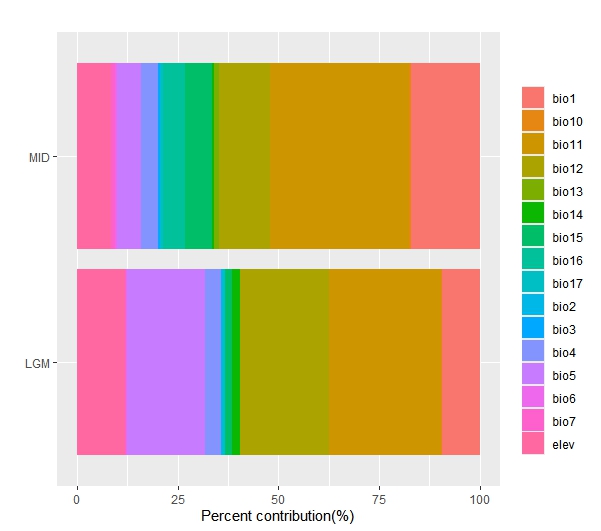

Supplement: Supplementary Figure 7 — The contributions of the corresponding variable were plotted from 16 factors that affected the distributions of two periods. [file Image7.jpeg]

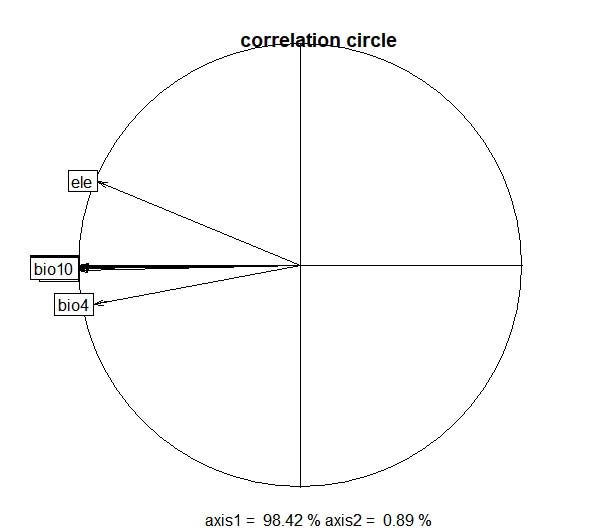

Supplement: Supplementary Figure 8 — Principal component analysis (PCA) in the ecological niche of 20 environmental factors. [file Image8.jpg]
